# Editorial: Virtual presence: loneliness, technology and the production of human (dis)connectedness

**DOI:** 10.3389/fdgth.2024.1445568

**Published:** 2024-07-09

**Authors:** Gemma Hughes, Lars E. F. Johannessen, Erik Børve Rasmussen

**Affiliations:** ^1^ School of Business, University of Leicester, Leicester, United Kingdom; ^2^ Nuffield Department of Primary Care Health Sciences, University of Oxford, Oxford, United Kingdom; ^3^ Department for Social Work, Child Welfare and Social Policy, Oslo Metropolitan University, Oslo, Norway

**Keywords:** loneliness, social isolation, technology—assistive/supportive, technology—ICT, telepresence, digital health

**Editorial on the Research Topic** Virtual presence: loneliness, technology and the production of human (dis)connectedness

This Research Topic brings together novel empirical and theoretical work on the relationship between loneliness, technology, and human (dis)connectedness. Our intellectual starting point is the dichotomy of technology as both a potential cause of and a remedy for loneliness. Technology is frequently critiqued for fostering only “artificial” connections, thereby undermining “real” interpersonal relationships (1, 2). At the same time, we are seeing technologists, industry designers, and the general public increasingly considering technology as means to enhance social connectedness and “tackle” loneliness (3).

The enduring tension in this dichotomy is a central theme of the research project, “*Virtual presence: A cultural analysis of the emergence of telepresence technologies as a solution to loneliness*”, from which the idea for this Research Topic emerged. *Virtual Presence* received funding just before COVID-19 was declared a global pandemic. Loneliness was already emerging as a significant policy and public health issue, with the idea of a “loneliness epidemic” in circulation (4). Interest in the topic exploded during the pandemic and the concomitant lockdown. During this time, more people than ever were using technology to achieve human connection, in new ways and in different settings, making this area of inquiry increasingly central to understanding health and digital health.

**Figure 1:**
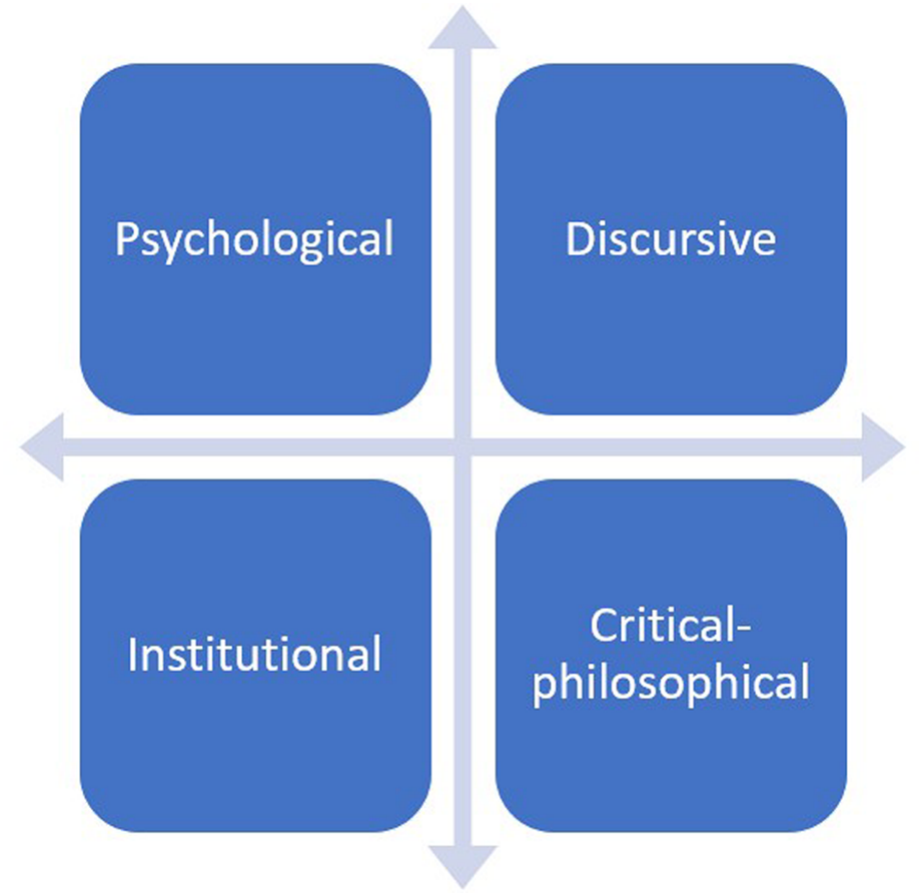
Approaches to questions of loneliness, technology and human (dis)connectedness.

In curating this collection, we have emphasized the principle of *pluralism*, highlighting multiple perspectives rather than confining research to isolated academic silos. The Research Topic offers four analytically distinct approaches to questions of loneliness, technology, and human (dis)connectedness (see Figure 1).

The first approach is *psychological*, treating loneliness as a subjective state that can be positively or negatively influenced by technology. This is the dominant approach in loneliness research (5) and informs four of the articles in this collection. The article by Akhtar used longitudinal survey data to examine whether the use of novel communication technology can increase social connectedness among nursing home residents in Norway. The author's results are largely positive, implying that the intervention has the potential to increase social connectedness and can be a feasible strategy for staying connected to the world outside of care homes. Drawing on survey data from Ireland during the pandemic, Fahy and Barry found that social capital is a key factor in enabling people to benefit from online communication (including social media, messaging applications and online groups) in terms of mental wellbeing and alleviating loneliness. Ling et al. used interview data to explore the experiences of US older adults using social technology during the early stages of the pandemic. The authors found that while the majority were highly motivated to use technology to maintain relationships during this period, their outcomes varied with differences in skills and access—highlighting the importance of the “digital divide” (6) in research on loneliness and technology. Finally, Mejova and Lu analyzed self-disclosures of loneliness on Twitter, finding that these surged dramatically during the first two months of the pandemic and that there was a gendered aspect to these expressions.

Moving away from the notion of loneliness as a measurable condition, the second approach shifts attention to how the relationship between loneliness, technology and (dis)connectedness is constituted *discursively* in policy, marketing, and various other contexts. This approach is exemplified in the work of Hughes et al, who used qualitative methods to explore the meanings of loneliness and social isolation as articulated in relation to the introduction of two telepresence technologies in the UK between 2020 and 2022. Their analysis revealed that loneliness is no longer seen merely as a subjective painful experience, but as a social and policy issue that requires resolution. Similarly, Jentoft analyzed UK loneliness policy between 2017 and 2021, focusing on how policy and political discourses on loneliness intersect with issues of technology and aging. The author argued that dominant discourse tends to focus on seemingly cost-effective technologies, sidestepping discussions about the broader societal factors that influence disconnected communities.

The third approach is *institutional*, shifting the emphasis from experiences or representations of loneliness to the institutional contexts in which people attempt to adopt technologies for social connection. Although this approach is reflected in several articles, it is most prominently illustrated in the work of Spoden and Ema, who investigated the implementation of telepresence robots for children and young people who are unable to attend school. By comparing the implementation processes in Japan and Germany, their study revealed how the potential for connection and inclusion through the use of such robots is influenced by a variety of structural, cultural, financial, and legal factors. This underscores the significance of how issues of loneliness, technology, and inclusion are navigated within highly institutionalized settings like schools.

The fourth approach is *critical-philosophical* and delves into deeper ontological, epistemological, and political questions about loneliness, technology, and (dis)connectedness. This approach is particularly prominent in Jacobs' work. Unlike other papers in this collection that focus on technologies *through* which people communicate, Jacobs examined technologies *with* which people communicate, in the form of “AI companions” that use artificial intelligence to form social relationships with human users. Drawing on an understanding of loneliness as “a significant change of meaningful relatedness”, Jacobs warns against seeing AI companions as potent solutions for loneliness; instead, they are likely to offer new ways of being lonely. The implications of producing new forms of human connection include the production of new forms of disconnection.

A pluralist reading of the relationship between loneliness and technology allows us to consider issues of loneliness as multiple; loneliness can be understood simultaneously as a measurable and malleable experience and as a cultural and epistemic category. Taking a pluralist approach allows for an understanding of how loneliness is interrelated with health through material and social resources; how the problem of loneliness is discursively constituted; and how new experiences of loneliness can arise despite, or even as a consequence of, new forms of human connection. Looking ahead, we encourage more research that adopts, and adds to these approaches, to interrogate the many overlaps and frictions between competing understandings and experiences of loneliness and technology.
